# *BvHP4b* gene in red beet promotes tuber enlargement and enhances resistance to Pst DC3000

**DOI:** 10.1186/s12864-025-11864-8

**Published:** 2025-08-07

**Authors:** Xiaozheng Xing, Zengyuan Tian, Shuran Yang, Xiaomin Wang, Zhiyang Xing, Di Wu, Shuting Zheng, Yuqi Guo

**Affiliations:** 1 School of Life Sciences, Zhengzhou University, Kexue Road 100, Zhengzhou, 450001 China; 2 School of Agriclutural Sciences, Kexue Road 100, Zhengzhou, 450001 China

**Keywords:** Red beet, *BvHP4b*, Disease resistance, Tuber enlargement

## Abstract

One important component of the cytokinin signaling pathway is the histidine phosphotransferase (HP), which plays an essential role in regulating downstream gene expression by transferring phosphate groups. The role of histidine phosphotransfer proteins (HPs) in sugar beets under abiotic and biotic stresses remains unclear. The results showed that the expression of *BvHP4b* in red beets increased in response to the exogenous hormone 6-BA. Transgenic Arabidopsis seedlings with heterologous expression of *BvHP4* T3 lines (*BvHP4*-OE) displayed enhanced growth and resistance to Pst DC3000. Moreover, the expression of NPR1 and PRs was increased in the T3 transgenic lines compared to the control. Subcellular localization analysis revealed that BvHP4b primarily localized to the cell membrane. Transgenic red beets overexpressing *BvHP4b* (*BvHP4b*-OX lines) and *bvhp4b* mutant plants (KO lines) using CRISPR/Cas9 technology were generated respectively. The *BvHP4b*-OX lines showed enhanced taproot development, which was attributed to the altered expression of *BvXTH33*, *BvCESA6*, *BvCDC2*, and *BvCEL1* genes. These lines also exhibited increased tolerance to Pst DC3000 by regulating synthesis of SA and modulating the expression of genes involved in plant immunity. In contrast, the KO plants were more susceptible to Pst DC3000 compared to the control plants. Furthermore, BvHP4b was shown to interact with BvCDC2 both *in* *vivo* and *in* *vitro*. Overall, this study suggests that the BvHP4b protein acts as a positive regulator, enhancing taproot enlargement and tolerance to Pst DC3000 in sugar beet plants.

**Supplementary Information:**

The online version contains supplementary material available at 10.1186/s12864-025-11864-8.

## Introduction

*Pseudomonas syringae* is a common plant pathogen that destroys plant cell membrane structure by secreting effector proteins or toxins, interferes with water and nutrient transport, inhibits mineral absorption, and ultimately leads to delayed plant growth and development [1]. Infected plants typically exhibit localized leaf necrosis spots, wilting, or stem ulcers, and in severe cases, can delay flowering and reduce fruit yield. This type of disease not only causes direct economic losses, but also increases planting costs due to prevention and control measures.

Plants have evolved a dual layer immune system consisting of pattern triggered immunity (PTI) and effector triggered immunity (ETI) to respond to pathogen threats. Plants perceive pathogen associated molecular patterns (PAMPs) through pattern recognition receptors (PRRs) on their cell membranes. When PRR (such as FLS2 or EFR) recognizes PAMP, it activates downstream signaling pathways and induces the NADPH oxidase RBOHD to catalyze the production of reactive oxygen species (ROS) on the cell membrane [2, 3]. RBOHD mediates the accumulation of ROS in the extracellular space by converting superoxide anions (O₂·⁻) into hydrogen peroxide (H ₂O ₂), thereby triggering a calcium signaling cascade and disease resistance gene expression [4]. When the pathogen effector protein breaks through PTI, plants recognize the effector protein through nucleotide binding oligomerization domain - leucine rich repeat receptor (NLR), activating ETI [5]. ETI typically triggers hypersensitivity reactions (HR) and systemic acquired resistance (SAR), significantly enhancing immune strength. In tomato (*Solanum lycopersicum*), pathogen infection can induce NLR protein to release its post transcriptional inhibition of NLR genes by inhibiting the expression of miR482, thereby amplifying disease resistance signals [6–8]. This negative feedback regulation mechanism ensures the dynamic balance of immune input and growth and development in plants under resource constraints.

Meanwhile, salicylic acid leaves are cellular signaling molecules involved in regulating plant defense against pathogens. The site of pathogen infection synthesizes salicylic acid glucoside (SAG), which is hydrolyzed by β -glucosidase to form free SA. SA promotes its monomerization by binding to NPR1 protein (dependent on cytoplasmic reduction state), and after being transported to the nucleus, interacts with TGA transcription factors to activate gene expression such as PR1, PR2 (β−1,3-glucanase), and PR5 (sweetener like protein), specifically resisting living nutritional pathogens [9, 10]. PR2 degrades fungal cell wall β−1,3-glucan [11]; PR3 (alkaline chitinase) and PR4 (containing Barwin domain) inhibit hyphal growth by binding to fungal chitin [12]; PR5 destroys the integrity of the pathogen membrane. The SA signal is synthesized through the isobranched acid synthase (ICS) pathway [13, 14], and the absence of its core regulatory factor NPR1 (such as the Arabidopsis * npr1* mutant) completely blocks PR1 expression [15], while overexpression of NPR1 significantly enhances broad-spectrum resistance.

Cytokinin plays a crucial role in regulating both development and responses to the environment. Cytokinin binding to histidine kinase receptor (AHK2/3/4) triggers autophosphorylation, and the phosphate group is transferred to B-type response regulatory factor (B-ARR) through AHP protein. Activated B-ARR enters the nucleus and regulates the expression of target genes (such as *ICS1*) by binding to the promoter region, promoting salicylic acid (SA) synthesis and activating defense genes such as *PR1* [16]. The *Arabidopsis thaliana* *ahk2 hk3* double mutant exhibits significantly lower resistance to Pseudomonas syringae than the wild-type Col-0 due to a deficiency in cytokinin signaling, which hinders SA accumulation [17]. B-ARR (such as ARR2) enhances the SA signaling pathway and amplifies PR1 expression by directly interacting with the bZIP transcription factor TGA3 and synergizing with NPR1 [18, 19]; A-ARR (such as ARR4) weakens immune responses by inhibiting B-ARR phosphorylation or competing for DNA binding sites [20]. Auxin promotes pathogen susceptibility by inhibiting the SA signaling pathway, while cytokinin significantly enhances resistance to Pst DC3000 by antagonizing auxin signaling (such as downregulating AUX/IAA genes) and activating SA synthesis [21].

The two-component system (TCS) is a transmembrane signal transduction system found in bacteria and plants. In bacteria, TCS included a membrane-associated histidine protein kinase (HK) and a cytoplasmic response regulator (RR) with a receiver (REC) domain. HK proteins primarily sense signals and autophosphorylation occurs in the histidine residue (H) of the HK domain. The phosphate is subsequently transferred to an aspartate residue (D) in the REC domain of the RR protein [22]. In eukaryotes, such as yeast and plants, TCS have evolved a more complex multi-step TCS signaling system with additional His phosphotransfer (HP) proteins, which contains an XHQXKGSSXS motif and are responsible for the transfer of phosphates between HKs and RRs, thereby further increasing the accuracy of its regulation [23]. HPs are the downstream target proteins of cytokinin receptor HKs, which receive and transfer the phosphate groups from the receptor HKs and transfer the phosphate groups to the downstream Arabidopsis response regulators (ARRs) to complete the downstream transduction of cytokinins.

The HP protein plays a role in salt and drought stress in rice [24], and in Arabidopsis [25]. Overexpression of the cucumber *CsHP1* gene triggers the expression of genes related to the auxin pathway [26–28]. Cytokinin can regulate the root meristematic tissue by inducing the expression of ARR genes, such as SHY2/IAA3, which inhibits ARF activity and PIN protein expression, leading to a redistribution of auxin [29, 30]. It has been found that the level of cytokinin is positively correlated with the formation and enlargement of adventitious roots [31]. 6-BA also plays an important role in promoting primary root enlargement. Based on the database, six HP genes were found in red sugar beets. In this study, qRT-PCR was used to analyze the gene expression patterns of red sugar beets treated with exogenous 6-BA and untreated. The results showed that the expression levels of *BvHP4a* and *BvHP4b* genes were highest after treatment with different concentrations of 6-BA. Therefore, these two genes were selected for subsequent functional studies.

Beet is a member of the *Amaranthaceae family*, known for its high nutritional content and medicinal properties. To investigate the function of the HP gene in beet, this study cloned the *BvHP4a* and *BvHP4b* genes from beet and generated transgenic *Arabidopsis thaliana* plants with ectopic expression of *BvHP4a* and *BvHP4b*, as well as beet plants overexpressing the *BvHP4b* gene. Additionally, gene editing techniques were employed to obtain mutant plants. Comparative analysis of taproot development and plant phenotype among transgenic plants, mutants, and control plants exposed to Pst DC3000 revealed the significant role of *BvHP4b* in both beet root development and resistance to Pst DC3000.

## Materials and methods

### Plant materials, growth conditions and treatments

The wild-type Arabidopsis used in the experiment is the Colombian ecological type, red beets are Detroit red beets preserved in the laboratory and cultivated in greenhouses.The seeds of *Arabidopsis thaliana* and red beet seeds *(B.vulgaris var.crucenta*) were sterilised by dipping in ethanol (75%) for 1 min and successively in NaClO (5%) solution for 10 min and then washed at least 5–10 times with sterilized water. Then the seeds were placed on Murashige and Skoog (MS) agar medium for germination. They were transplanted into pots containing sterilised perlite and vermiculite for 15 days with 8 h light/16 h dark at 28 °C after they grew until five leaves stage. The uniform seedlings were treated with 10 mg/L, 15 mg/L and 20 mg/L 6-BA.

### Construct a phylogenetic tree and Bioinformatics analysis of cis-acting elements of *HP4b* genes

We conducted a BLAST search on the Ensembl Plants online website by using the amino acid sequence of BvHP4b as the query. We identified six BvHP genes, named BvHP1a, BvHP1b, BvHP1c, BvHP4a, BvHP4b, and BvHP6, respectively. To further analyze the evolutionary relationships of these genes, we used MEGA11 software to construct a phylogenetic tree using maximum likelihood (ML) method. During the construction process, we chose the JTT + G (Jones Taylor Thornton + Gamma) model to consider the rate heterogeneity of amino acid substitution and conducted 1000 boostrap replicates to evaluate the reliability of the branches.For analysis on cis-acting elements of *BvHP* genes, a 2000 bp region located upstream of the translational start site of each *BvHP* gene was submitted to the online PlantCARE database (http://bioinformatics.psb.ugent.be/webtools/plantcare/html/) to predict putative cis-elements.

### Analysis on expression pattern of *BvHP* genes in red beet under treatment with 6-BA

Red sugar beet plants were treated with 6-BA when they reached the 4–6 leaf stage. The concentrations of 6-BA were 10, 15, and 20 mg/L, and the control group was treated with distilled water. Every 5 days, the red sugar beet is treated by spraying leaves and irrigating roots. Each plant is sprayed with 50 mL of leaves until liquid droplets drip down, ensuring that the red sugar beet leaves are completely soaked. Then, 50 mL is used to irrigate roots for a total of 5 treatments. Extract sugar beet RNA and store it in a −80 ℃ freezer for directed reverse transcription into cDNA, followed by qRT-PCR analysis. Total RNA was extracted by TRIzol reagent (Invitrogen, USA) according to the manufacturer’s instructions. The integrity and quality of total RNA were examined by NanoDrop1000 spectrophotometer and agarose gel electrophoresis. First-strand cDNA was synthesized using a random hexamer primer. The qRT-PCR (Table 1) was conducted with Maxima SYBR Green Master Mix (Thermo Scientific) using a Real-time Quantitative PCR System (iQ5, Bio-Rad, USA) with three repetitions. *BvActin* (OX392556.1) was used as reference gene. The relative expression data were analyzed using the 2 ^− ΔΔCT^ method.

**Table 1:** Primers used for analysis on expression in *HP*gene family in red beet

Gene	primer	sequence
*BvActin*	Fp	5'-TGCTTGACTCTGGTGATGGT-3'
	Rp	5'-AGCAAGATCCAAACGGAGAATG-3'
*Bv-9g224770(BvHP4a)*	Fp	5'-TCCACCCACACCCTCCTATC-3'
	Rp	5'-TTCTTTGGGCAGGGAACACT-3'
*Bv-7g165880(BvHP1a)*	Fp	5'-AACCAAAGCTCTCATCGGCG-3'
	Rp	5'-ACGGGTTTCCAAGGTCTAATCC-3'
*Bv-9g219550(BvHP6)*	Fp	5'-ATCCTTCAAAACGGGTCTTCC-3'
	Rp	5'-AAGTCCGGAGAACTCTCATCT-3'
*Bv-4g083230(BvHP1b)*	Fp	5'-CAGCAGTTCCAGCATCGGT-3'
	Rp	5'-AGGAATTGATCCACCGGCT-3'
*Bv-2g032730(BvHP4b)*	Fp	5'-AGTAGCTCAAGCATCGGAGC-3'
	Rp	5'-TCGCAGGAGACGACAAACAT-3'
*Bv-5g114260(BvHP1c)*	Fp	5'-GCATTAGGGTCCTCCGATCC-3'
	Rp	5'- TTTACCACCACCCCCGTTTC-3'

### Construction of plant vector, *Agrobacterium*-mediated transformation and regeneration of transgenic *Arabidopsis* plant and transgenic red beet

The CDS sequence of *BvHP4a* and *BvHP4b* were cloned by PCR using a Mastercycler ^®^nexus-PCR Thermal Cycler (Eppendorf, Germany) with primers (*BvHP4a*: Forward primer 5′-CACCTTTTGCACTGATGGAGCGTAAC-3′ and Reverse primer 5′-CGCCGCCGAGTAGCCATCTCCCTCGG-3′; *BvHP4b*: Forward primers 5′-CACCAAGCCGTGTCTGTTAAGACT-3′ and Reverse primer 5′- AAAATAAGCCTCAAGTTTCTTTCT-3′). The PCR amplification program was conducted as follows: 95℃ for 2 min, 35 cycles of 94℃ for 30 s, 55℃ for 30 s, and 72℃ for 2 min, followed by a final extension of 72℃ for 5 min. The PCR products of *BvHP4a* and *BvHP4b* gene were inserted into a T-vector and was sequenced by the Sangon Biotech Co.,Ltd.(Shanghai, China). The CDS sequence without stop codon were inserted into entry plasmid (Invitrogen, USA), then cloned into a transformation vector pMDC83 using the Gateway Technology (ClonaseTM Kit, Invitrogen, USA). The resulting vector 35 S:: *BvHP4a*-*GFP* and 35 S:: *BvHP4b*-*GFP*, were introduced into *Agrobacterium tumefaciens* strain GV3101 by the liquid nitrogen freeze-thaw method. Arabidopsis plants were transformed with floral dipping technique (Clough and Bent 1998). And red beet were transformed with meristematic callus tissue following the method of Gurel et al. [32]. Select plump red sugar beet seeds, gently tap open the red sugar beet seed shells to select intact seeds, disinfect with 75% ethanol for 30 s, disinfect with 5% NaClO for 10 min, and rinse with sterile water 6–7 times. The sterilized seeds are placed in germination medium and cultured under light to obtain sterile seedlings. Cultivate sterile red sugar beet seedlings to two true leaves, select sweet cabbage cotyledons for Agrobacterium transformation, then inhibit the cultivation of sterile red sugar beet seedlings to two true leaves, select sweet cabbage cotyledons for Agrobacterium transformation, and finally obtain resistant seedlings through antibacterial cultivation and hygromycin screening. After refining and transplanting, overexpressed *BvHP4b* gene red sugar beet was obtained.

*Transgenic Arabidopsis* plants and red beet plants were screened on MS agar medium containing 40 mg/L hygromycin to obtain transgenic lines. Homozygous T3 transgenic plants were harvested and confirmed first by PCR using the specific forward and reverse primer which were designed for fused gene (forward primer for *BvHP4a*/*BvHP4b* and reverse primer for *GFP*). Genomic DNA was extracted from leaves of 6-week-old seedling leaves of T3 transgenic plants and wild-type plants by CTAB. PCR was performed to identify transgenic plants expressing *BvHP4a* using primers BvHP4a-FP 5’-CACCTTTTGCACTGATGGAGCGTAAC-3’ and GFP-RP (5’-ACGGGAGAAGCACTGCACGC-3’; and *BvHP4b* with primers BvHP4b-FP: 5’-CACCAAGCCGTGTCTGTTAAGACT-3’’andGFP-RP5’-ACGGGAGAAGCACTGCACGC-3’. The amplification was performed using the program: 94 ºC for 2 min, followed by 35 cycles of program (94 ºC for 30 s, 56 ºC for 30 s, 72 ºC for 2 min), and 72 ºC for 5 min. And the confirmation for transgenic lines were performed again by Western Blot methods [33].

### Western blotting analysis

Total protein was extracted from WT and T3 transgenic Arabidopsis seedlings and red beet using RIPA Lysis and Extraction Buffer (Thermo Scientific™) and quantified using the BCA Protein Assay Kit. Protein samples (200 µg) were electrophoresed in 10% SDS-PAGE and gels were transferred to nitrocellulose membranes. The membranes were blocked with TBST buffer (10 mM Tris/HCl (pH 7.5), 150mM NaCl and 0.05% Tween-20) supplemented with 5% non-fat milk for 2 h and incubated with primary antibodies (Anti-GFP antibody, abcam, diluted at 1:200) in TBST buffer with 5% BSA overnight at 4 °C. Afterwards, the membranes were washed three times (10 min each) with TBST buffer and incubated with the secondary antibodies (Goat Anti-Mouse IgG H&L (HRP), abcam, dilution at 1:1000) for 2 h. After being washed three times with TBST buffer, the membranes were incubated with a chromogenic agent Enhanced HRP-DAB Chromogenic Substrate Kit (Boster).

### Subcellular localization assay

The CDS (without the stop codon) of *BvHP4a* and *BvHP4b* were obtained by PCR using *BvHP4a*−1300-F and *BvHP4a*−1300-R or *BvHP4b*−1300-F and *BvHP4b*−1300-R as primers containing specific restriction sites *KpnI* and *XbaI* (Table 2). Then, it was inserted into the pCAMBIA1300 to obtain recombination vector pCAMBIA1300-*BvHP4a*-*GFP* and pCAMBIA1300-*BvHP4b*-*GFP*. Then recombination vector pCAMBIA1300-BvHP4a-GFP or pCAMBIA1300-*BvHP4b*-*GFP* and control pCAMBIA1300-*GFP* were introduced into *Agrobacterium tumefaciens* strain GV3101. After injection into *N.benthamiana* leaves with GV3101containing recombination vector, the protoplasts of tobacco leaves were observed for transient expression of GFP using confocal fluorescence microscopy.

**Table 2:** Primers for subcellular localization of BvHP4a and BvHP4b

Gene	Primer	sequence
BvHP4b-1300	Fp	5’-atacaccaaatcgactctagaAAGCCGTGTCTGTTAAGACTTGTCT-3’
	Rp	5’-gcccttgctcaccatggtaccAAAATAAGCCTCAAGTTTCTTTCTCA-3’
BvHP4a-1300	Fp	5’-atacaccaaatcgactctagaTTTTGCACTGATGGAGCGTAAC-3’
	Rp	5’-gcccttgctcaccatggtaccACGCCGCCGAGTAGCCAT-3’

### CRISPR/Cas9 vector construction and generation of *bvhp4b* KO mutant plants

To generate red beet lines with mutations in the *BvHP4b* gene, two guide RNAs targeting different exons regions of *BvHP4b* were designed using online tool by http://skl.scau.edu.cn/ (Table 3). The designed CRISPR/Cas9 plasmid containing the two sgRNA cassette was inserted into pHSN401 to obtain recombinant vector pSHN401-*BvHP4b*. The constructed vector was transformed into *Beta vulgaris* callus tissue by *A. tumefaciens* GV3101 mediated transformation [34]. Genomic regions flanking the target site of the gene *BvHP4b* in transgenic T0 red beet plants were amplified with primers and sequenced. The sequenced DNA obtained from transgenic plant lines was aligned with the *BvHP4b* sequence using NCBI BLAST.

**Table 3:** gRNA sequence for tar*ge*ting *BvHP4b*

gRNA	Primer sequence
*BvHP4b*-DT3-BsF	5'-atatatggtctcgattGCCAGTTCCTCAAAATCTAGgtt-3'
*BvHP4b*-DT3-F0	5'-tGCCAGTTCCTCAAAATCTAGgttttagagctagaaatagc-3'
*BvHP4b*-DT4-R0	5'-aacTTCAGATGATAAAGCAAGACaatctcttagtcgactctac-3'
*BvHP4b*-DT4-BsR	5'-attattggtctcgaaacTTCAGATGATAAAGCAAGACaa-3'

### Mutant identification

*Arabidopsis* mutants (SALK_051911C) were identified using the three primer method. Use Lp, Rp, and Bp primers to amplify the target gene and identify the *hp4b* Arabidopsis mutant (Table 4).

**Table 4:** Mutant identification primers

Primer	sequence
Bp	5'-ATTTTGCCGATTTCGGAAC-3'
Lp	5'-ATCTCGACGAACAATTCATGG-3'
Rp	5'-AAAATTCGACGACGATTCCTC-3'

### Transcriptional activation and Y2H assay

We predicted the genes that may have protein interactions with BvHP4b through the String website and constructed an interaction network. We used ZDOCK to score and screen the predicted genes that interact with BvHP4b, and found that BvCDC2 has a strong interaction with the target BvHP4b. Confirm the interaction by screening the inferred interaction partner BvCDC2 through Y2H analysis. The ORF of *BvHP4b* and *BvCDC2* gene were cloned with primer pairs with EcoRI and BamHI restriction site (Table 4). The amplified PCR products was then ligated into the pGBKT7 vector (bait vector). Similarly, *BvCDC2* gene was amplified using *BvCDC2*-pGADT7-F and *BvCDC2*-pGADT7-R primers with *EcoRI* and *BamHI* restriction site and ligated into the pGADT7 vector(prey vector) (Table 5). The recombinant vectors pGBKT7-*BvHP4b* + pGADT7; pGBKT7 + pGADT7-*BvCDC2* were co-transformed into yeast competent cells (*Sacccharomycees cerevisiae*) (Y2HGold) respectively following the manufacturer’s instructions (Zoman, Beijing). Then the transformed mixture were spread on SD/-Leu/-Trp and SD/-Leu/-Trp/-His/-Ade media to observe colony growth for estimation the self-activation of the *BvHP4b* and *BvCDC2*. After excluding self-activation, the fusion vectors pGBKT7-*BvHP4b* and pGADT7-*BvCDC2* were co-transformed into yeast cell to validate the interaction between *BvHP4b* and *BvCDC2*.

**Table 5:** Primers for Yeast two-hybrid assays

Gene	Primer	sequence
pGBKT7-*BvHP4b*	Fp	5'-gtaccagattacgctcatatgAAGCCGTGTCTGTTAAGACTTGTCT-3'
	Rp	5'-atgcccacccgggtggaattcAAAATAAGCCTCAAGTTTCTTTCTCA-3'
pGADT7-*BvCDC2*	Fp	5'-tcagaggaggacctgcatatgATGGACCAGTATGAAAAAGTTGAGAA-3'
	Rp	5'-tcgacggatccccgggaattcGGGTACAAAGCCAATGTCCTTG-3'

### Bimolecular fluorescence complementation (BiFC) assay

The PCR products of *BvHP4b* (excluding the stop codon) was amplified by PCR using primers pXY104-BvHP4b-F and pXY104-BvHP4b-R. The amplified sequence was then ligated to the vector pXY104, resulting C-terminal fragment of the YFP (pXY104- -cYFP). Similarly, the *BvCDC2* sequence (including the stop codon) was amplified by PCR using primers pXY106-BvCDC2-F and pXY106-BvCDC2-R. The amplified sequence was then inserted into frame of N-terminal fragment of YFP (pXY106-nYFP). The pXY104- BvHP4b and the pXY106-BvCDC2 recombinant vector were co-transformed into *Agrobacterium* (GV3101) by means of *Agrobacterium*-mediated infiltration. The mixture containing *Agrobacterium* cells were injected tobacco leaves according to methods [35]. Finally, The YFP fluorescence of protoplasts from tobacco leaves is observed using fluorescence confocal microscopy with 514 nm excitation (ZEISS).

### Bacterial cultivation, inoculation, pathogenicity assays

*Pseudomonas syringae pv. tomato DC3000* (Pst DC3000) was cultivated as previously described [36]. Bacterial cells were rinsed with water, diluted in 10 mM MgCl_2_ (OD600 = 0.002), then vacuum-infiltrated into *Arabidopsis* or red beet leaves of plants to examine resistance-related gene expression and SA accumulation.

Five samples of four leaf discs (1 cm diameter) were taken from three plants at 2 h after inoculation (Day 0 and 2 days), and homogenized in 1 ml of 10 mM MgCl_2_ to determine bacterial populations via serial dilution plating.

### Measurement of endogenous Salicylic acid content

The content of plant SA in transgenic A. thaliana seedlings and red beet expressing *BvHP4b* was measured using ELISA kits. Endogenous hormones were extracted according to a modified method described by Chakraborty et al. Specifically, 0.1 g of plant tissues of each sample were ground with a mortar and pestle at liquid N2 in 0.9 mL of 50 mM PBS buffer. The extract was then centrifuged at 4000 rpm for 15 min and the supernatant was used to detect the concentration of SA according to the instructions on the ELISA kit. A standard curve was obtained using standard SA at different known concentrations (3, 6, 12, 24, and 48 pmol/L) and the concentrations of SA produced were calculated by comparison with the standard curve.

### RT-PCR analyses on expression of genes involved resistance to Pst DC3000 and enlargement of taproot

After the *Arabidopsis thaliana* and red beet were inoculated with Pst DC3000, and total RNA extraction was performed following the TRIzol Reagent (Invitrogen) instruction. The extracted RNA was then reverse transcribed into cDNA to use as a template for qRT-PCR analysis (1 µg). Specific primers for disease-related genes (*NPR*1, *PR*s) and tuber development (*BvCEL1*, *BvCESA6* and *BvXTH33*) in red beet were utilized to detect their expression (Table 6). Actin for *Arabidopsis thaliana* and red beet were used for normalization.

**Table 6:** RT-PCR Primers for genes involved in disease resistance and tuber development

Gene	primer	sequence
*AtActin*	Fp	5'-ATGAAGGACTGGAGAGGTGGA-3'
	Rp	5'-CGTTAAGAGCTGGAAGCACC-3'
*AtPR1*	Fp	5'-AGCGATGTTTACGAACCCCA-3'
	Rp	5'-CCAGGCTAAGTTTTCCCCGT-3'
*AtPR2*	Fp	5'-ATGGATCACCGAGAAGGCCA-3'
	Rp	5'-TGGGTCTCACATTCCCTAAGC-3'
*AtPR3*	Fp	5'-TCTGGCAAACGCTACTACGG-3'
	Rp	5'-ACGTCCACACTCCAATCCAC-3'
*AtPR4*	Fp	5'-CAACAATGCGGTCGTCAAGG-3'
	Rp	5'-CACCCTTAAACACTTGCCGC-3'
*AtPR5*	Fp	5'-GCTGGGGATAAGACCATCCG-3'
	Rp	5'-CCGGTACAAGTGAAGGTGCT-3'
*BvActin*	Fp	5'-TGCTTGACTCTGGTGATGGT-3'
	Rp	5'-AGCAAGATCCAAACGGAGAATG-3'
*BvXTH33*	Fp	5'-CCCTGGTTTCACCTCTGGTATT-3'
	Rp	5'-CTCTTCTCTCCCTGTCTTTACACT-3'
*BvCEL1*	Fp	5'-CCGTGATTCCTGGTGTTGCT-3'
	Rp	5'-TGGCACTTGGTGATTGGAGT-3'
*BvCESA6*	Fp	5'-GCCTGTGATGTTTGTGCCTT-3'
	Rp	5'-GGGGGACTACCTTTCTGCTT-3'

### Protein interaction prediction

The output of protein interaction prediction included PIPER pose energy and PIPER pose score, which are shown in the Project Table (Table 7). PIPER represents the interaction energy between two proteins with the following formula: E = W_1_ *E_rep_ + W_2_ *E_attr_ + W_3_ *E_elec_ + W_4_*E_DARS’,_ Where w, indicates weights, Erep and Eatt indicate the repulsive and attractive contributions to the van der Waals interaction energy, and Elec is an electrostatic energy term. This sum is reported as PIPER pose energy. EDARs is a pairwise structure-based potential constructed by the ‘decoys as the reference state’ (DARS) approach, and it primarily represents desolvation contributions. The fourth term (weighted) is reported as the PIPER pose score [37].

**Table 7:** Combining pattern analysis

**7XQK_A ** **Residues**	**4EUK_A ** **Residues**	**Distance (Å)**	**Specific Interactions**	**#HB**	**#Salt Bridges**	**Surface ** **Complementarity**	**7XQK_A Buried SASA(%)**	**4EUK_A Buried SASA(%)**
A: LYS 906	B: GLU 784	1.8	1x hb, 1x salt bridge to B: GLU 784	1	1	0.65	61.4	100
A: LYS 906	B: ASP 828	2	1x hb, 1x salt bridge to B: ASP 828	1	1	0.8	61.4	95.8
A: ASP 901	B: ARG 844	2.2	1x hb, 1x salt bridge to B: ARG 844	1	1	0.83	1.3	79.9
A: GLY 899	B: ARG 844	1.9	2x hb to B: ARG 844	2	0	0.73	62.5	79.9
A: ASN 898	B: ARG 841	2.2	1x hb to B: ARG 841	1	0	0.93	81.1	32.5
A: GLU 890	B: CYS 830	2.1	1x hb to B: CYS 830	1	0	0.74	86	37
A: MET 883	B: MET 827	2	1x hb to B: MET 827	1	0	0.86	99.8	100
A: MET 883	B: VAL 829	2.4	1x hb to B: VAL 829	1	0	0.86	99.8	96.9
A: ILE 881	B: VAL 825	1.9	1x hb to B: VAL 825	1	0	0.84	99.9	100
A: ILE 881	B: MET 827	1.8	1x hb to B: MET 827	1	0	0.81	99.9	100
A: PRO 879	B: VAL 825	2.1	1x hb to B: VAL 825	1	0	0.87	55.6	100
A: LEU 878	B: ARG 844	2	2x hb to B: ARG 844	2	0	0.81	89.2	79.9
A: LEU 878	B: GLU 847	1.9	1x hb to B: GLU 847	1	0	0.78	89.2	56.3
A: GLY 147	B: GLN 799	1.8	1x hb to B: GLN 799	1	0	0.54	0	100
A: ARG 50	B: GLN 799	1.9	1x hb to B: GLN 799	1	0	0.65	0	100
A: THR 14	B: SER 795	1.8	1x hb to B: SER 795	1	0	0.46	60.5	95.2

### Data analysis

All experiments were performed in triplicate, and the results were presented as mean ± standard error of the mean (SEM). Statistical analysis of experimental data was subjected to analysis of variance using one-way ANOVA, and statistical differences between control and treated groups were determined (*P < 0.05; **P < 0.01; ***P < 0.001).

## Results

### Expression analysis of *BvHP* genes in response to 6-BA

**Fig. 1:**
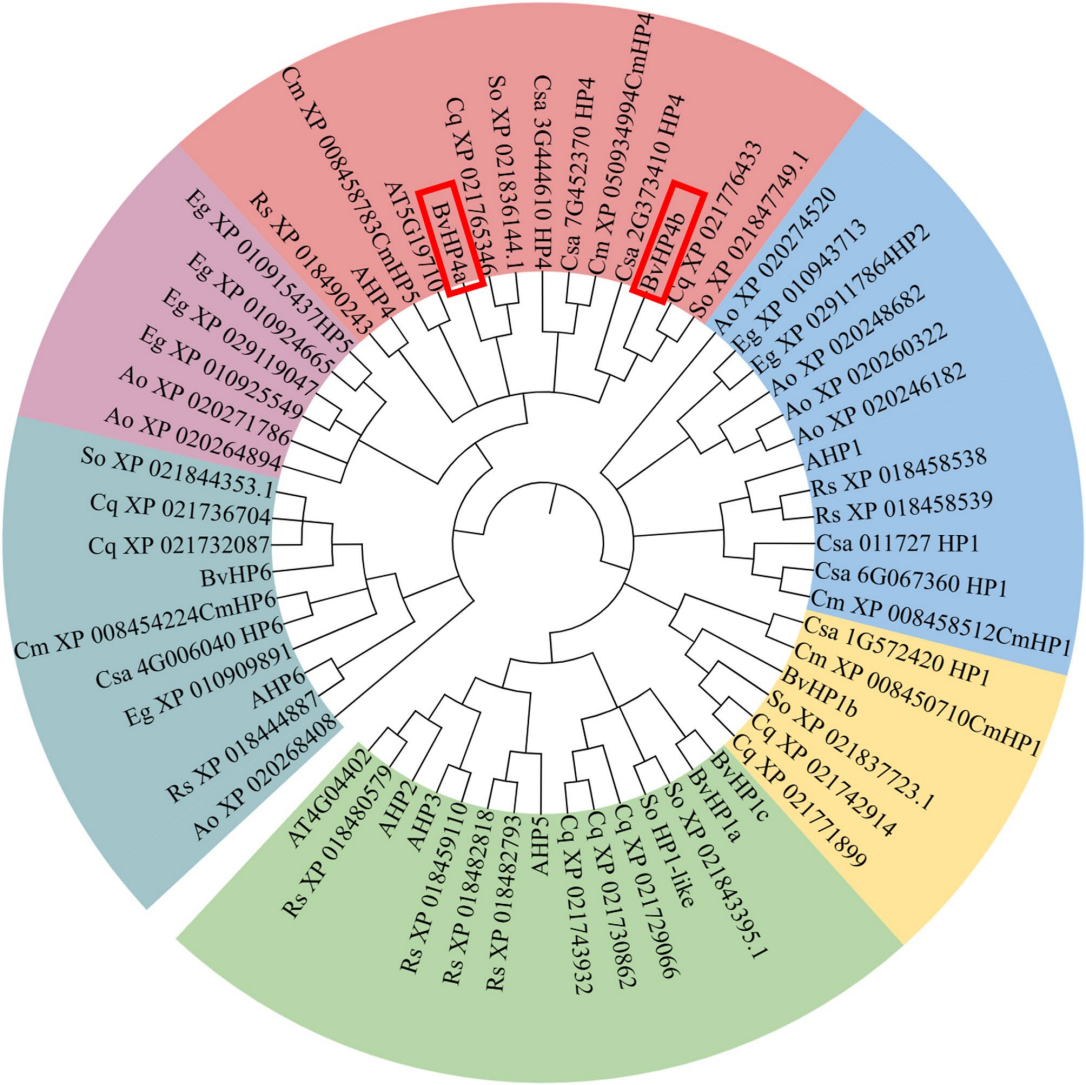
Introduction of the HP gene family in red sugar beet, Arabidopsis thaliana, spinach, cantaloupe, cucumber, oil palm, asparagus, quinoa, and radish Transform the tree. (ML)

A phylogenetic tree was constructed using 63 members of the *HP*s gene family from 9 species including red sugar beet, *Arabidopsis thaliana*, spinach, cantaloupe, cucumber, oil palm, asparagus, quinoa, and radish. The results showed that the HP family proteins of these 9 species can be divided into 5 major subgroups. BvHP4 has the closest genetic relationship with the HP4 genes of spinach and quinoa, and is more closely related to the HP4 genes of cucumber, melon, and radish. (Fig. 1).

**Fig. 2:**
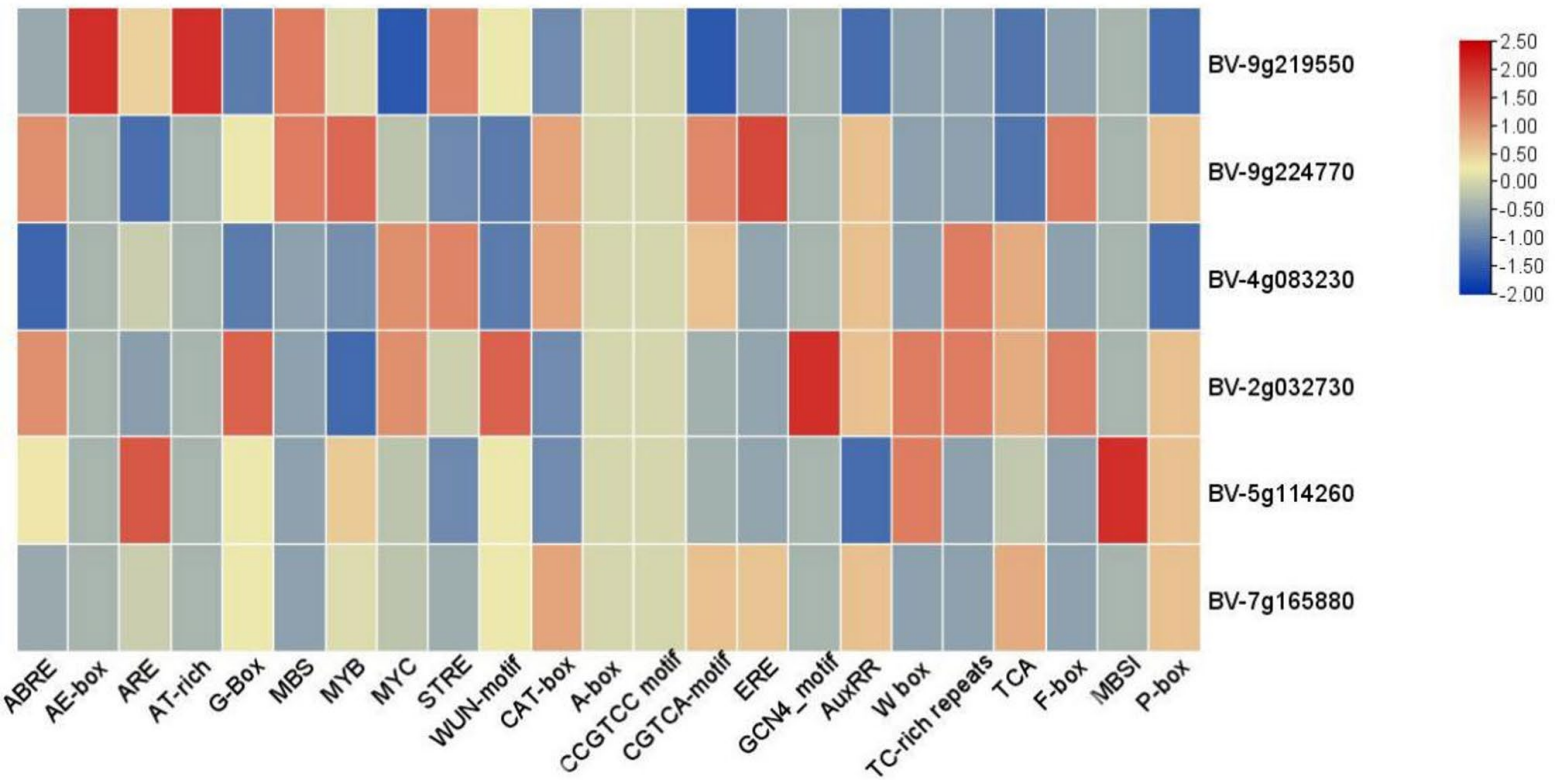
Cis-acting elements in promoter of the HP gene family in red beets

Cis-acting element analysis revealed that the promoter contains various plant hormone responsive elements, including F-box and meristematic expression element CAT-box. Additionally, it contains defense and stress response elements such as TC-rich repeats, drought stress response element MYB-binding sites involved in drought inducibility (MBS), salicylic acid response element TCA, and wound response element WUN (Fig. 2). These findings suggest that the HP gene is involved in multiple transcriptional regulatory processes in red beet.

**Fig. 3:**
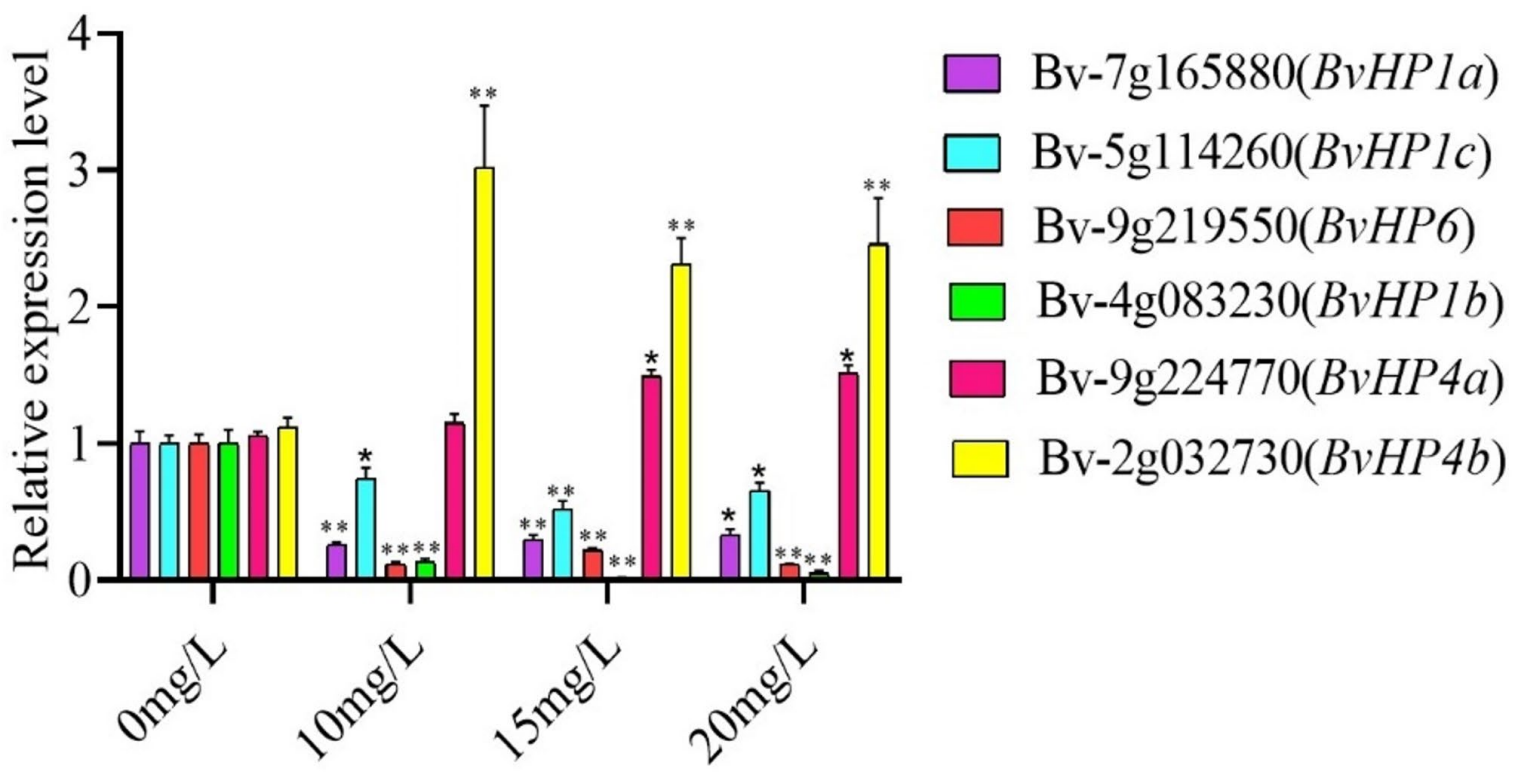
Expression pattern of the sugar beet *HPs* gene family under the treatment with 6-BA. (t test, *n* = 3, *:*P* < 0.05, **:*P* < 0.01)

In response to 6-BA, the *BvHP* genes were differentially expressed, and mRNA abundance of *BvHP4a* and *BvHP4b* was the highest at different concentration of 6-BA compared with other *BvHP*, specially at 10 mg/L 6-BA (Fig. 3).

### Generation of transgenic *Arbidopsis* plants ectopic expressing *BvHP4a* and *BvHP4b* and transgenic red beet plants overexpressing *BvHP4b*

**Fig. 4:**
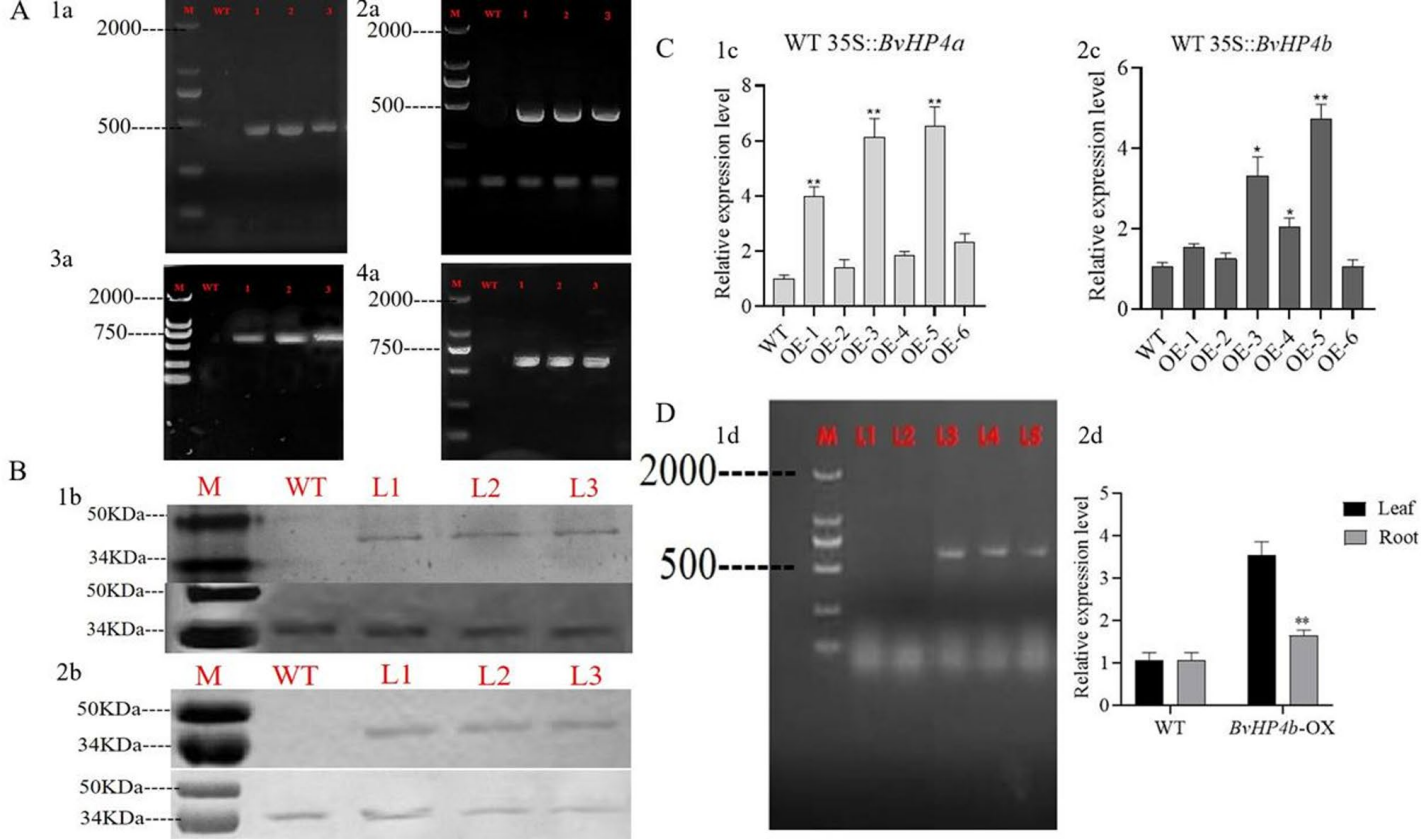
Confirmatin of transgenic plants. **A** Gene amplification of *BvHP4b *(Fig. 1a) or *BvHP4a *(Fig. 2a). PCR for fused gene with forward primer of *BvHP4a* (a3) or *BvHP4b* (a4) and reverse primer for GFP. **B** Protein expression of transgenic *Arabidopsis* ectopic expression *BvHP4b* (1b) and *BvHP4a* (2b) by Western Blot. Bands of GFP- GAPDH as reference protein (2b). **C** RT-PCR analysis of transgenic *Arabidopsis* lines *BvHP4a*-T_3_(1c) and *BvHP4b*-T_3_(2c). **D** Confirmation of transgenic red beet overexpressing *BvHP4b* with PCR (L1 and L2: WT; L3, L4, and L5: transgenic red beet lines). (1d). The expression of *BvHP4b* in transgenic beets(2d). Data are presented as mean ± SE. (t test, *n* = 3, *: *P* < 0.05, **: *P* < 0.01)

We obtained transgenic *Arabidopsis thaliana* and red beet with expressing *BvHP4a* and *BvHP4b* through *Agrobacterium* mediated dipping method. Select well grown *Arabidopsis thaliana* leaves for DNA extraction and use them as templates for identification. Firstly, PCR was performed using forward and reverse primers of *BvHP4b* or *BvHP4a* to identify the *BvHP4b* or *BvHP4a* of *transgenic Arabidopsis* seedlings *BvHP4a*-OE (Fig. 4A 1a) and *BvHP4b*-OE (Fig. 4A 2a). Then, integrated *BvHP4b-GFP* genes or *BvHP4a-GFP* genes were identified using forward primers of *BvHP4a* or *BvHP4b* and reverse primers of GFP. As seen in Fig. 4A 3a and Fig. 4A 4a, no band in WT and one band in L1 to L3, which corresponded to non transgenic seedlings of *Arabidopsis *(WT) and transgenic seedlings *BvHP4a*-OE L1-3 and *BvHP4b*-OE L1-3. Western blot technology was used to verify whether the *BvHP4a* GFP and *BvHP4b* GFP fusion proteins were expressed in transgenic plants. The molecular weight of the BvHP4a-GFP is 44 kDa, the molecular weight of the BvHP4b-GFP fusion protein is 45.2 kDa (refer to Figure S1), and the molecular weight of the internal reference protein GADPH is 36 kDa. The results are shown in Fig. 4B, indicating that expression of the BvHP4a-GFP and BvHP4b-GFP genes (Line1-3). This result showed that the exogenous BvHP4a and BvHP4b genes were successfully transferred into wild-type plants, resulting in transgenic Arabidopsis.

Quantitative reverse transcription PCR (qRT-PCR) was used to validate the expression of BvHP4a and BvHP4b genes in different T3 transgenic Arabidopsis lines. As seen in Fig. 4C, the expression levels of OE-1, OE-3, and OE-5 lines in *BvHP4a* transgenic *Arabidopsis* were relatively high, and *BvHP4b* in the OE-3, OE-4, and OE-5 lines of *BvHP4b*-OE transgenic *Arabidopsis* were higher than those in other transgenic plants. The seeds of these three lines were selected for subsequent experiments.

### Ectopic expression of *BvHP4b *and *BvHP4a* enhanced growth and development of *Arabidopsis* plants; Overexpression of *BvHP4b* promoted enlargement of tuberous root

**Fig. 5:**
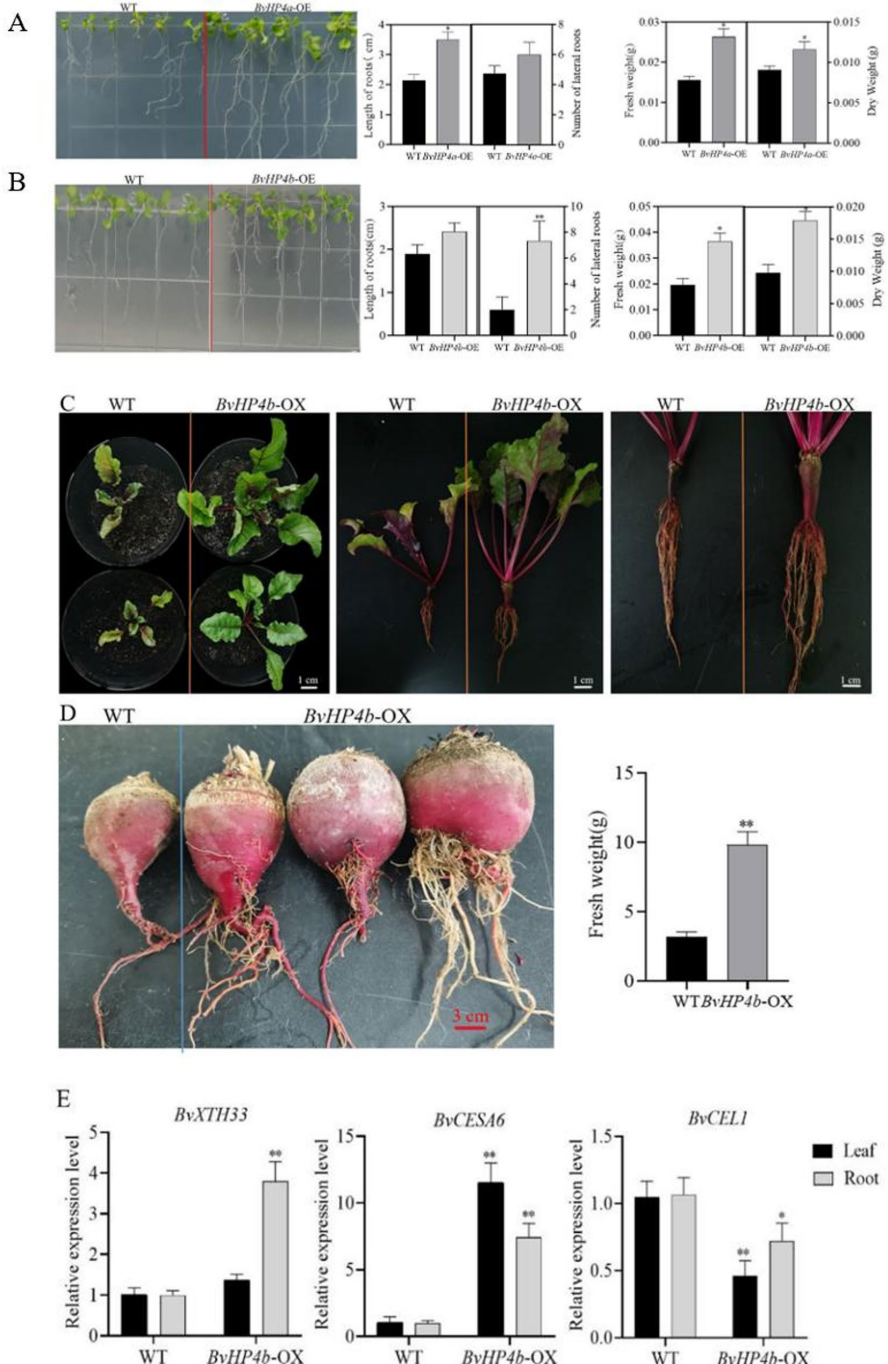
Growth of WT and transgenic plants. **A** The length of primary root, number of lateral root, fresh weight, and dry weight of transgenic Arabidopsis lines *(BvHP4a-OE*). **B** The length of primary root, lateral root number, fresh weight, and dry weight of transgenic Arabidopsis lines *(BvHP4b-OE*). **C** Phenotype of *BvHP4b-OX* transgenic red beet grown for 10 weeks. **D** The tuberous roots of transgenic red beet (*BvHP4b-OX*) grown for 5 months. **E** Analysis of gene expression levels related to cell wall synthesis in leaves and roots in WT and *BvHP4b-OX*. (OE: Arabidopsis and OX: Beta vulgaris) Data is presented as mean ± SE. (t test, *n* = 3, *:*P* < 0.05, **:*P* < 0.01)

Our results showed that the length of main roots, the number of lateral roots, the fresh weight and dry weight in transgenic *Arabidopsis* ectopic expressing *BvHP4a* was higher than WT by 64.32%, 68.42%, 68.56% and 83.76%, respectively than WT (Fig. 5A); The main root length, the number of lateral roots, the fresh weight and dry weight in transgenic Arabidopsis ectopic expressing *BvHP4b* was higher by 27.6%, 126.6%, 97.60% and 82.65%, respectively, than WT (Fig. 5B).

The tuberous root in red beet plants overexpressing *BvHP4b*, which were grown for 10 weeks, were larger than that of the control. The plant height and the size of tuberous root are significantly higher than those of control (Fig. 5C). After grown for 5 months, it was evident that the fresh weight of tuberous root in *BvHP4b*-OX plants is higher than that of control, displaying a significant enlargement (Fig. 5D). The expression of *BvHTH33* and *BvCESA6* in BvHP4b-OX were significantly increased in the leaves and tuberous roots, while *BvCEL1* decreased obviously. (Fig. 5E) It was inferred that the *BvHP4b* gene affects the growth of red beets by regulating synthesis of cell wall composition.

### The *BvHP4b*-gene-editing plants by CRISPR/Cas9 technology

**Fig. 6:**
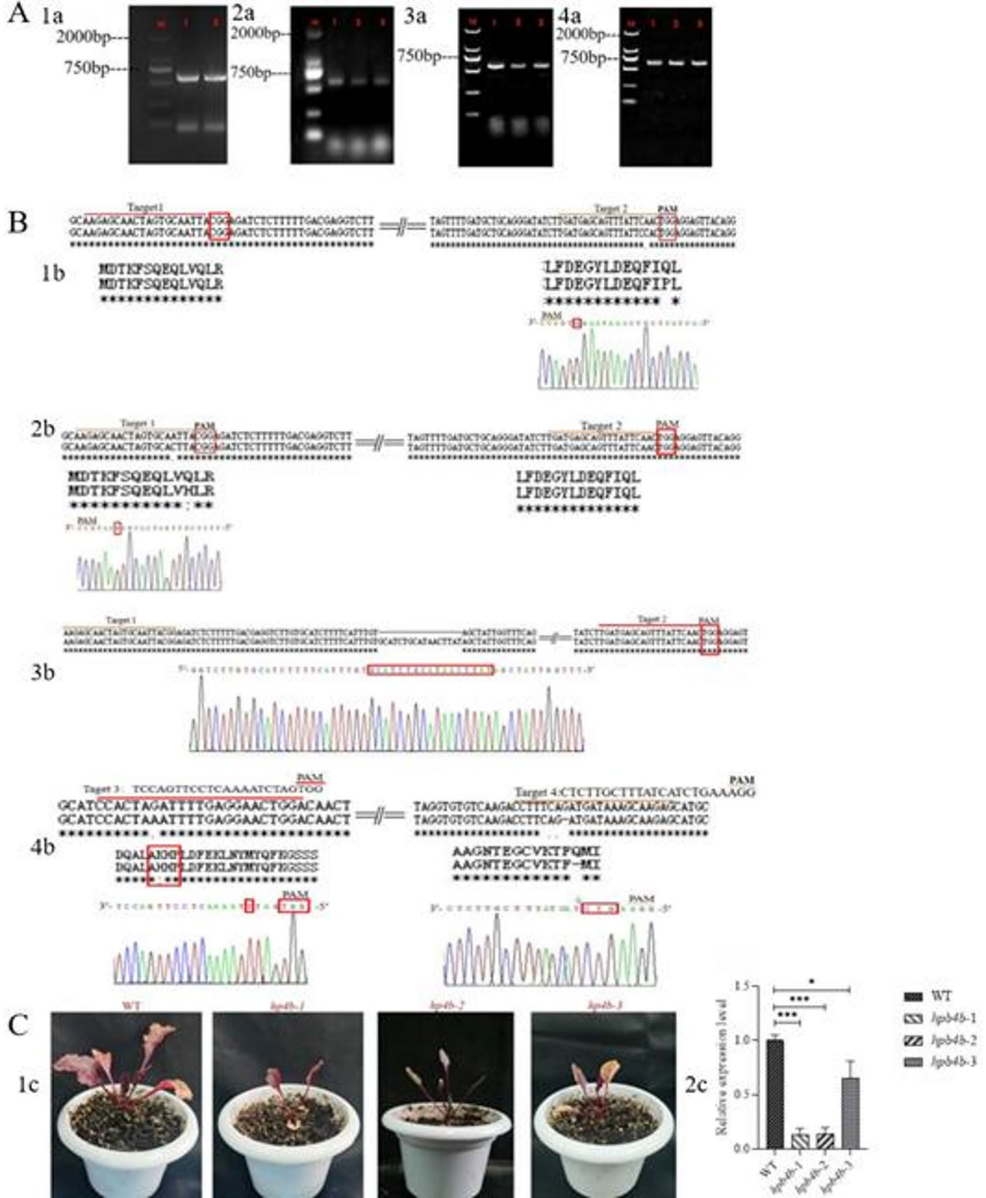
CRISPR/Cas9 technology for mutated with *HP4b* in red beet. **A** pSHN401-*BvHP4b* vector construction. PCR of sgRNA Box (Target 1 and 2) (1a). Identification of Escherichia coli by PCR (Target 1 and 2) (2a). PCR of sgRNA Box (Target 3 and 4) (3a). Identification of Escherichia coli by PCR (Target 3 and 4) (4a). **B** Mutation site with *BvHP4b* gene in red beet. **C** Phenotype of *bvhp4b* mutant lines (1c). Expression analysis of the *BvHP4b* gene in mutant plants (2c). Data are shown as mean ± standard error (t test, *n* = 3, *:*P* < 0.05, **:*P* < 0.01, ***:*P* < 0.001)

Two independent guide sgRNAs (single guide RNA) for different regions of *BvHP4b* (Targets 1 and 2) were designed to target *BvHP4b* (Fig. 6B) using the online tool CRISPR-P (http://cbi.hzau.edu.cn/crispr/). To assemble the two gRNAs in PHSE, the two target sites were incorporated into PCR forward and reverse primers, respectively. The PCR fragment was purified after running PCR using pCBC-DT1DT2 plasmid as template. The purified PCR fragments (T1T2-PCR) was then subjected for assembly into PHSE by the same restriction ligation reaction. The constructed vectors were verified by enzyme digestion and sequencing [38]. Primers used are listed in Table 2. The CRISPR/CAS9 plasmid (pHSN401) carrying the two sgRNA cassettes (Fig. 6 1a) was transformed into red beet by Agrobacterium-mediated transformation to obtain mutagenesis of *BvHP4b* in transgenic red beet.

After transformation, we obtained a total of 3 transgenic lines with mutagenesis at target sites 1 and 2, while only 1 line with mutagenesis at target 3 and 4. As shown in (Fig. 6B), in the mutagenesis at target site 1 and 2, base substitution was detected, resulting in changes in the encoded amino acids (Fig. 6 1b, 2b). There was one mutant line with a mutagenesis of insertion of base downstream target 1, (Fig. 6 3b). In the plants targeted site 3 and site 4, both target sites were mutated simultaneously, with base substitution and base deletion (Fig. 6 4b). These results demonstrate that mutated lines with *BvHP4b* gene (KO, designated as *bvhp4b-1*, *bvhp4b-2 bvhp4b-3*, and *bvhp4b*) were successfully obtained in sugar beets using CRISPR/Cas9 technology (T0 generation).

The expression level of the *bvhp4b-1*, *bvhp4b-2* and *bvhp4b-3* lines was measured using qRT-PCR. The results confirmed that expression of *bvhp4b* in KO lines were lower in comparison to control red beet (Fig. 6 2c). The plant height and number of leaves of T_0_ lines *bvhp4b-1*, *bvhp4b-2* and *bvhp4b* lines were lower than control plant (Fig. 6 1c).

### The *BvHP4b* and *BvHP4a* proteins are located on the cell membrane

**Fig. 7:**
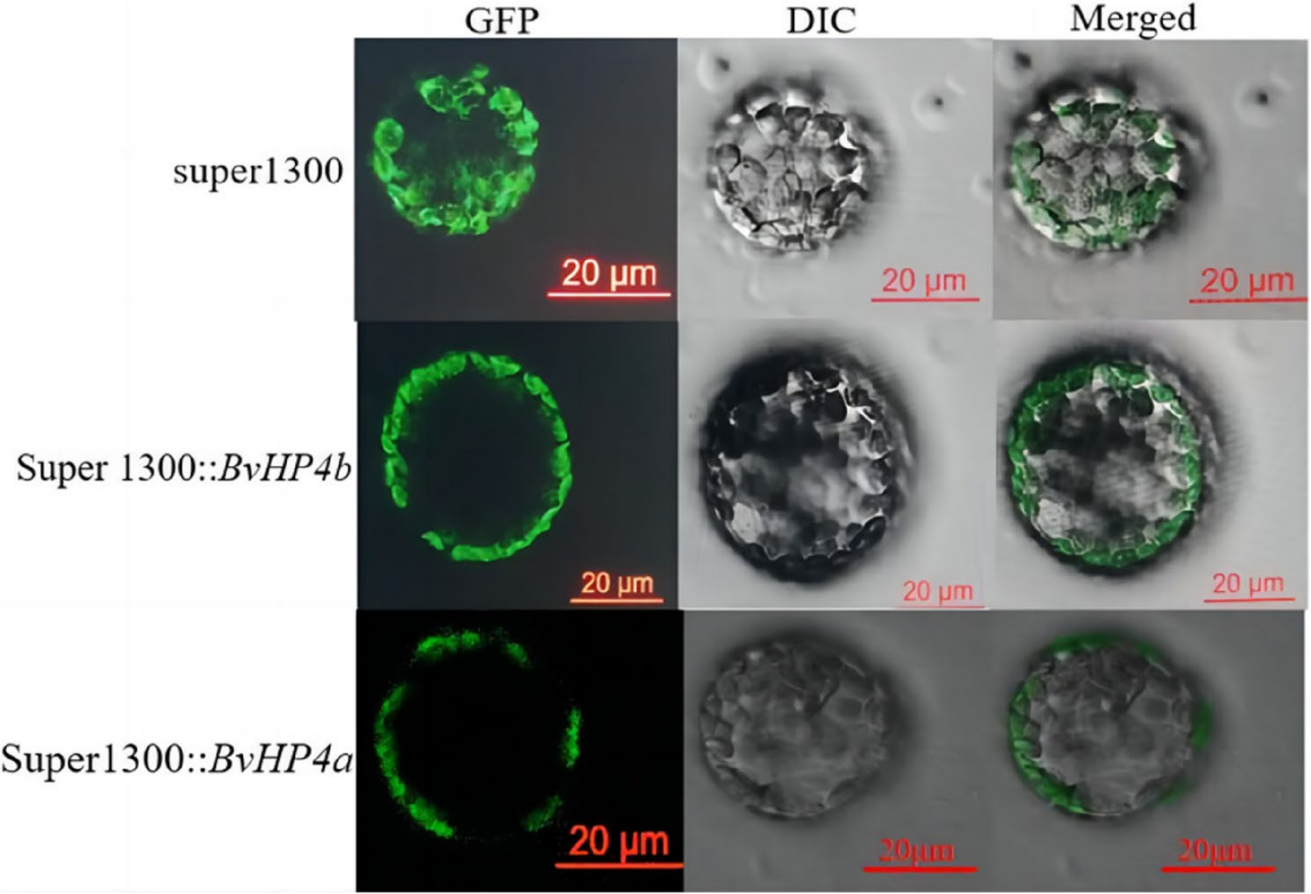
Subcellular localization of*BvHP4a* and *BvHP4b* in tobacco protoplasts using laser confocal microscopy

As shown in the results showed in Fig. 7, the Fused *BvHP4a*-GFP and *BvHP4b*-GFP protein was expressed in cell membrane, which suggested that *BvHP4a* and *BvHP4b* are localized on the cell membrane.

### *BvHP4b* gene enhances resistance against the pathogenic bacteria Pst DC300

**Fig. 8:**
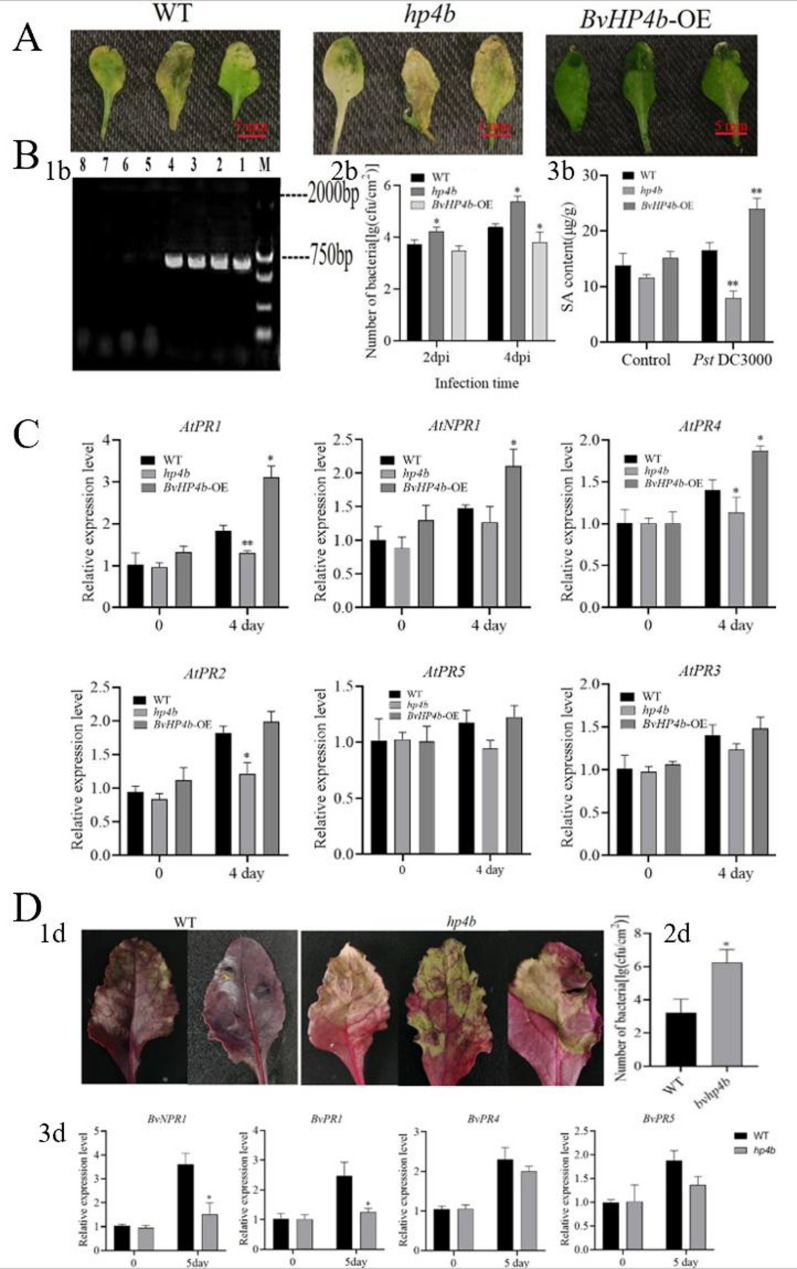
Arabidopsis

Because transgenic *Arabidopsis BvHP4b*-OE grew better that *BvHP4a*-OE, we selected *BvHP4b*-OE for further assay on its resistance to Pst DC3000. Homozygous *athp4b* mutant plants(SALK_051911C) was identified in Fig. 8 1b. PCR product of lane1-4 using primer BP and RP, lane 5–8 using RP and LP. After inoculation with Pst DC3000 for 4 days (4 dpi), *athp4b* mutant plants showed more susceptible than WT, while *BvHP4b*-OE plants did not show obvious impaired symptoms (Fig. 8A). The population of Pst DC3000 in *athp4b* mutant increased by 13.2%, and 22.7% compared to WT at 2 dpi and 4 dpi, respectively. The population in WT was higher by 15.1% than that in *BvHP4b*-OE at 4 dpi. (Fig. 8 2b). These results indicated that *BvHP4b* can enhance resistance to Pst DC3000 in *Arabidopsis*. As shown in Fig. 8 3b, there were no significant differences in salicylic acid levels among the wild type, *athp4b* mutant, and *BvHP4b*-OE under non inoculation treatment. SA of the *BvHP4b*-OE increased by 44.28% compared with WT, however, SA of *athp4b* plants was decreased by 85.88% compared to WT, at 4 dpi. Compared to the WT, the expression levels of *NPR1*, *NPR2*,*PR1*,*PR*4 and *PR5* genes in *BvHP4b*-OE were upregulated, while down-regulated in the *athp4b* compared to WT at 4 dpi (Fig. 8C). Specially, expression of *AtNPR1*,*AtPR1*,*AtPR4* were upregulated significantly.

Similarly, the mutated sugar beet plants was more susceptible to Pst DC3000 at 2 dpi and 5 dpi (Fig. 8 1 d), while the control plants had no obvious impaired symptoms, and the population of the *Bvhp4b* plants was significantly higher than that of control at 5 dpi (Fig. 8 3 d). Compared to the control, there was a significant decrease in the expression levels of *BvNPR1* and *BvPR1* genes in *bvhp4b* plants at 5 dpi (Fig. 8 3 d).

### *BvHP4b* interact with *BvCDC2* proteins

**Fig. 9:**
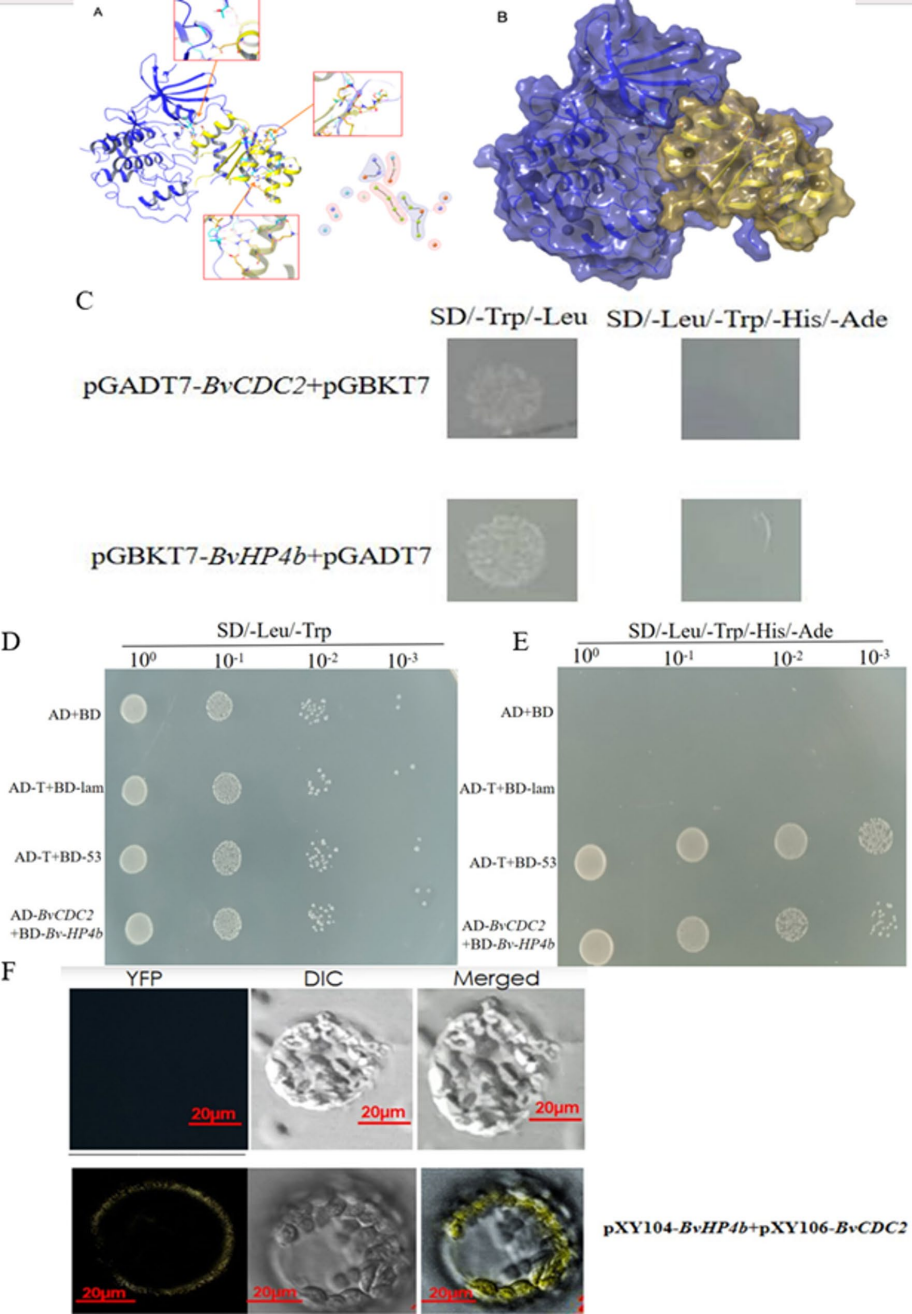
Protein interaction prediction: **A **The protein marked with blue and yellow indicates BvCDC2, and BvHP4b, respectively. The hydrogen bonds, and the represents salt bridge interactions were indicated with yellow color and red dashed line in the figure. **B **Three dimensional structural diagram of interacting proteins. The pTM value is 0.8. **C** self-activation verification results.** D** Yeast cells were grown on the following selective media SD/-Leu/-Trp. **E** Yeast cells were grown on media SD/-Leu/-Trp/-His/-Ade. Yeast cells harboring the pGAD-T7+pGBKT7-53 served as the positive control. Yeast cells harboring the pGAD-T7+pGBKT7-Lam served as the negative control. **F **Bimolecular fluorescence complementation assay on interaction between BvHP4b and BvCDC2

The interaction network was constructed using the STRING website (https://string-db.org/), and the predicted interacting proteins with *BvHP4b* were then evaluated using ZDOCK(http://zdock.umassmed.edu/). A schematic diagram of two protein docking was generated using Pymol software, (Fig. 9B) predicted protein interaction docking using Schrodinger software, and calculated a protein docking affinity of −645.231 kcal/mol (Fig. 9A).

Based on target protein with strong interaction with *BvHP4b*, *BvCDC2* was selected for confirmation to interact with *BvHP4b* in vitro and in vivo with Y2H and BiFC assay, respectively. As seen in Fig. 9D and E, colonies of pGADT7-*BvCDC2* + pGBKT7 or pGBKT7-*BvHP4b* + pGADT7 successfully appeared on SD/-Leu/-Trp. However, they did not appear on selective media SD/-Ade/-His/-Leu/-Trp, indicating no self-activation of pGBKT7- *BvHP4b* (Fig. 9C). When the two recombinant vectors pGADT7-*BvCDC2* and pGBKT7- *BvHP4b* were co-transformed yeast cells, AH109, the colonies appeared on selective media SD/-Ade/-His/-Leu/-Trp. The gel electrophoresis results of successful carrier construction are shown in Figure S2. These results suggest an interaction between *BvHP4b* and *BvCDC2*.To further analyze the interaction between *BvHP4b* and *BvCDC2* proteins in vivo, the vectors for bimolecular fluorescence complementation (BiFC) analysis were constructed. After the two recombinant vectors pXY104-*BvHP4b* and pXY106-*BvCDC2* were introduced into protoplast of tobacco, YFP fluorescence protein was detected onto the cell membrane. As the negative control, protoplast of tobacco with construct of pXY104-nYFP(YN) and pXY106 had no fluorescence (Fig. 9F).

## Discussion

Cytokinin promotes cell division and plant growth [39]. During the process, Cytokinin transduction is mediated by continuous phosphate transfer. HP proteins allow plants to transport phosphate groups to perform multi-step phosphorelay [40]. Both *BvHP4a* and *BvHP4b* proteins are located on the cell membrane, which is consistent with the results of *Arabidopsis*.In this study, higher dry and fresh weights as well as larger taproot were obtained in the transgenic plants expressing *BvHP4a* and *BvHP4b*, indicating that *AHPs* genes play a positive regulatory role in the development of plants [41].

The enlargement of the taproot was due to an increases in cell number and cell volume [42]. During the elongation of the cells, there were many enzymes which were involved in the degradation of polysaccharides, such as celluloses, polygalacturonases, and xyloglucan endotransglycosylase/hydrolase in cell wall [43]. The *BES1* gene regulates cellulose synthesis by interacting with CESAs (Cellulose Synthases) [44]. Our results showed that the expression of this gene was significantly higher than that of the WT, both in the leaves and roots. Cellulose enzymes can hydrolyze cellulose in the cell wall into cellobiose and glucoseADDIN. Here, the expression of the *BvCEL1 *(Cellulosome) gene in transgenic sugar beet was reduced, indicating that the *BvHP4b* gene may promote cell wall synthesis by increasing cellulose content. Xyloglucan is heteropolysaccharide in the primary cell wall of plants and is major component of hemicellulose. XTH (Xyloglucan endotransglucosylase/hydrolase) catalyzes the hydrolysis or transfer of xyloglucan molecules, leading to changes in cell wall structure. The expression of *BvXTH33* gene in leaves and taproots of the transgenic sugar beet was significantly higher than that in WT, indicating that BvHP4b may modulate change in cell wall structure, ultimately promoting taproot enlargement.

Previous report showed that the increased endogenous cytokinin levels in the overexpressing IPT plants lead to enhanced resistance against *Pst DC3000* [45], implying that Cytokinins play a role in plant immunity. During pathogen infection, cytokinins leads to the activation of HKs (histidine kinases) genes, *HP* (histidine phosphotransferase) genes, RR (response regulator), and PR-1(pathogenesis-related gene1), but the specific mechanism is not clear. Here, the cross-talk between the cytokinin and salicylic acid signaling pathways helps improve plant responses to pathogenic bacteria. The transgenic *Arabidopsis* showed significant disease resistance, while WT *Arabidopsis thaliana* showed susceptible to Pst DC3000. after inoculation. In the *BvHP4b*-OE, the expression of *NPR*1(non-effector of pathogenesis-related genes 1) and *PR*1 were significantly higher than those in the WT. The more content of salicylic acid were detected. Therefore, it can be inferred that the *BvHP4s* gene may enhance plant disease resistance by modulating the expression of defense genes in the salicylic acid (SA) signaling pathway.

The SA signal pathway plays a significant role in combatting pathogenic microorganisms in living organisms [46]. When plants are infected by pathogens, they synthesize salicylic acid glucoside (*SAG*) at the site of infection in response to the pathogen. This *SAG* later converts into SA as it reaches the target site and accumulates to a certain extent. Accumulated SA, in combination with SA binding protein (*SABP*), alters the redox potential of both the extracellular and intracellular environment, facilitating the binding of *NPR1* with the TGA transcription factor in the nucleus. This binding promotes the expression of SA-related PRs genes, leading to the activation of the plant’s defense response and improving its resistance to pathogens [9, 10]. When the *NPR1* gene in *Arabidopsis thaliana* is knocked out, expression of *PR1* is inhibited in response to pathogens. However, when *NPR*1 is overexpressed, *Arabidopsis* becomes more resistant to pathogens. PR genes are activated by SA and can protect plants against pathogens by degrading cell walls, breaking down toxins [15, 47]. PR-2, PR-3, and PR-4 proteins are involved in the hydrolysis of pathogenic microorganisms’ cell wall components, damaging the microorganisms that have entered the organs of plants.

*CDC2* (Cell division cycle gene 2), with a molecular weight of 34 kDa, can catalyze the phosphorylation of specific proteins. The *CDC2* protein, along with cyclin, forms a complex called maturation-promoting factor (MPF) that promotes the onset of mitosis during the M phase of cell division [31]. Overexpression of *AtCyCD2* in tobacco is found to shorten the duration of G1 phase and increase the rate of fast cell division [48]. *CDC2* is highly expressed in the root meristem zone of rice [49]. *GmCDC2-S5* is highly expressed in roots and rhizobia, while *CDC2-S6* is more active in aerial tissues [50]. Furthermore, the *CDC2* gene is the only *CDC* gene that functions during both the G1-S and G2-M phases of the cell cycle. In the G1 phase, upon stimulation by growth factors, *CDC2* collaborates with *CDK4* and *CDK6* to phosphorylate downstream proteins, thereby promoting the transcription of several genes. During the M phase, *CDC25* dephosphorylates *CDK*, removing the inhibitory phosphate, indicating the significant role of this gene in cell cycle progression [51]. Studies in *Arabidopsis* have shown that CDK8 promotes the expression of *NPR1* and *PR1*, playing a role in establishing plant immunity [11]. These results indicate that CDC genes not only regulate the cell division cycle but also promote plant immunity. In this study, *BvHP4b* was found to interact with BvCDC2, it is speculated that shortening the cell cycle may promote cell proliferation, increase the number and volume of cells, and ultimately lead to an increase in the weight of the main root. Additionally, this gene may help mitigate damage caused by Pst DC3000 by regulating SA signaling pathways, which ultimately mediate the expression of *NPR1* and *PRs*, leading to tolerance to Pst DC3000.

## Supplementary Information

Supplementary Material 1.

## Data Availability

Data availability statementThe original contributions presented in this study are included in the article. The specific gene sequences analyzed can be retrieved from the GenBank repository using accession numbers [XM_010695737.2] and [XM_010686495.2]. The corresponding protein sequence is available from the NCBI RefSeq repository under accession number [XP_010684797.1]. All data can be accessed directly through these accession numbers on the NCBI website: https://www.ncbi.nlm.nih.gov/. If you have further questions, please contact the corresponding author.

## References

[1] Savary S, Willocquet L, Pethybridge SJ, et al. The global burden of pathogens and pests on major food crops, vol. 3. Nature Ecology & Evolution; 2019. p. 430–9. 10.1038/s41559-018-0793-y 30718852

[2] Dangol S, Chen Y, Hwang BK, et al. Iron-and reactive oxygen species-dependent ferroptotic cell death in rice-magnaporthe oryzae interactions.[J]. Plant Cell. 2019;31(1):189–209. 30563847 10.1105/tpc.18.00535 PMC6391706

[3] Najafi J, Brembu T, Vie AK, et al. PAMP-INDUCED SECRETED PEPTIDE 3 modulates immunity in Arabidopsis[J]. J Exp Bot. 2020;71(3):850–64. 31665431 10.1093/jxb/erz482

[4] Tsuda K, Katagiri F. Comparing signaling mechanisms engaged in pattern-triggered and effector-triggered immunity[J]. Curr Opin Plant Biol. 2010;13(4):459–65. 20471306 10.1016/j.pbi.2010.04.006

[5] Zipfel C, Oldroyd GE. Plant signalling in symbiosis and immunity[J]. Nature. 2017;543(7645):328–36. 28300100 10.1038/nature22009

[6] Noutoshi Y, Okazaki M, Kida T, et al. Novel plant immune-priming compounds identified via high-throughput chemical screening target Salicylic acid glucosyltransferases in Arabidopsis[J]. Plant Cell. 2012;24(9):3795–804. 22960909 10.1105/tpc.112.098343 PMC3480303

[7] Canto-Pastor A, Santos B, Valli AA, et al. Enhanced resistance to bacterial and oomycete pathogens by short tandem target mimic RNAs in tomato[J]. PNAS. 2019;116(7):2755–60. 30679269 10.1073/pnas.1814380116 PMC6377479

[8] Quentin M, Allasia V, Pegard A, et al. Imbalanced lignin biosynthesis promotes the sexual reproduction of homothallic oomycete pathogens[J]. PLoS Pathog. 2009;5(1):e1000264. 19148278 10.1371/journal.ppat.1000264 PMC2613516

[9] Dong X. NPR1, all things considered[J]. Curr Opin Plant Biol. 2004;7(5):547–52. 15337097 10.1016/j.pbi.2004.07.005

[10] Li J, Brader G, Palva ET. The WRKY70 transcription factor: a node of convergence for jasmonate-mediated and salicylate-mediated signals in plant defense[J]. Plant Cell. 2004;16(2):319–31. 14742872 10.1105/tpc.016980 PMC341906

[11] Van Loon LC, Van Strien EA. The families of pathogenesis-related proteins, their activities, and comparative analysis of PR-1 type proteins[J]. Physiol Mol Plant Pathol. 1999;55(2):85–97.

[12] Selitrennikoff CP. Antifungal proteins[J]. Appl Environ Microbiol. 2001;67(7):2883–94. 11425698 10.1128/AEM.67.7.2883-2894.2001 PMC92957

[13] Spoel SH, van Ooijen G. Circadian redox signaling in plant immunity and abiotic stress[J]. Antioxid Redox Signal. 2014;20(18):3024–39. 23941583 10.1089/ars.2013.5530 PMC4038994

[14] Yang YN, Kim Y, Kim H, et al. The transcription factor ORA59 exhibits dual DNA binding specificity that differentially regulates ethylene- and jasmonic acid-induced genes in plant immunity[J]. Plant Physiol. 2021;187(4):2763–84. 34890461 10.1093/plphys/kiab437 PMC8644270

[15] Ding Y, Dommel MR, Wang C, et al. Differential quantitative requirements for NPR1 between basal immunity and systemic acquired resistance in *Arabidopsis thaliana*[J]. Frontier Plant Sci. 2020;11:570422. 10.3389/fpls.2020.570422 PMC7530841 33072146

[16] Argueso CT, Ferreira FJ, Epple P, et al. Two-component elements mediate interactions between cytokinin and Salicylic acid in plant immunity[J]. PLoS Genet. 2012;8(1):e1002448. 22291601 10.1371/journal.pgen.1002448 PMC3266875

[17] Choi J, Huh SU, Kojima M, et al. The cytokinin-activated transcription factor ARR2 promotes plant immunity via TGA3/NPR1-dependent Salicylic acid signaling in Arabidopsis[J]. Dev Cell. 2010;19(2):284–95. 20708590 10.1016/j.devcel.2010.07.011

[18] Naseem M, Wolfling M, Dandekar T. Cytokinins for immunity beyond growth, galls and green islands[J]. Trends Plant Sci. 2014;19(8):481–4. 24794463 10.1016/j.tplants.2014.04.001

[19] Pertry I, Vaclavikova K, Depuydt S, et al. Identification of Rhodococcus fascians cytokinins and their modus operandi to reshape the plant[J]. PNAS. 2009;106(3):929–34. 19129491 10.1073/pnas.0811683106 PMC2630087

[20] Synkova H, Semoradova S, Burketova L. High content of endogenous cytokinins stimulates activity of enzymes and proteins involved in stress response in Nicotiana tabacum[J]. Plant Cell Tissue Organ Cult. 2004;79(2):169–79.

[21] Naseem M, Kaltdorf M, Dandekar T. The nexus between growth and defence signalling: auxin and cytokinin modulate plant immune response pathways[J]. J Exp Bot. 2015;66(16):4885–96. 26109575 10.1093/jxb/erv297

[22] Mizuno T. Compilation of all genes encoding two-component phosphotransfer signal transducers in the genome of escherichia coli. DNA Res. 1997;4:161–8. 10.1093/dnares/4.2.161. 9205844 10.1093/dnares/4.2.161

[23] Huo RX, Liu ZN, Yu XL, Li ZY. The interaction network and signaling specificity of two-component system in Arabidopsis. Int J Mol Sci. 2020;21:4898. 10.3390/ijms21144898. 32664520 10.3390/ijms21144898 PMC7402358

[24] Sun L, Zhang Q, Wu J, et al. Two rice authentic histidine phosphotransfer proteins, OsAHP1 and OsAHP2, mediate cytokinin signaling and stress responses in rice[J]. Plant Physiol. 2014;165(1):335–45. 24578505 10.1104/pp.113.232629 PMC4012592

[25] Jeon J, Kim J. Arabidopsis response Regulator1 and Arabidopsis histidine phosphotransfer Protein2 (AHP2), AHP3, and AHP5 function in cold signaling[J]. Plant Physiol. 2013;161(1):408–24. 23124324 10.1104/pp.112.207621 PMC3532271

[26] Utsumi Y, Tanaka M, Utsumi C, et al. Integrative omics approaches revealed a crosstalk among phytohormones during tuberous root development in cassava. Plant Mol Biol. 2022:249–69. No.3. 10.1007/s11103-020-01033-8 32757126

[27] Chang L, Ramireddy E, Schmulling T. Lateral root formation and growth of Arabidopsis is redundantly regulated by cytokinin metabolism and signalling genes[J]. J Exp Bot. 2013;64(16):5021–32. 24023250 10.1093/jxb/ert291 PMC3830484

[28] Chang L, Ramireddy E, Schmulling T. Cytokinin as a positional cue regulating lateral root spacing in Arabidopsis[J]. J Exp Bot. 2015;66(15):4759–68. 26019251 10.1093/jxb/erv252 PMC4507779

[29] Matsuo T, Yoneda T, Itoo S. Identification of free cytokinins and the changes in endogenous levels during tuber development of Sweet-potato (Ipomoea-batatas lam)[J]. Plant Cell Physiol. 1983;24(7):1305–12.

[30] Tian Q, Uhlir NJ, Reed JW. Arabidopsis SHY2/IAA3 inhibits auxin-regulated gene expression[J]. Plant Cell. 2002;14(2):301–19. 11884676 10.1105/tpc.010283 PMC152914

[31] Moubayidin L, Perilli S, Dello IR, et al. The rate of cell differentiation controls the Arabidopsis root meristem growth phase[J]. Curr Biol. 2010;20(12):1138–43. 20605455 10.1016/j.cub.2010.05.035

[32] Gurel S. Sand-wounding of shoot and petiole explants enhances transformation efficiency in sugar beet (Beta vulgaris L.) by Agrobacterium-mediated transformation. Sugar Tech. 2021(No.2).

[33] Tian Z, Chen Y, Chen S, et al. AcdS gene of Bacillus cereus enhances salt tolerance of seedlings in tobacco (Nicotiana tabacum L.). Biotechnol Biotechnological Equip. 2022:902–13. No.1.

[34] Kim WN, Kim HJ, Chung YS, Kim HU. Construction of multiple guide RNAs in CRISPR/Cas9 vector using Stepwise or simultaneous golden gate cloning: case study for targeting the FAD2 and FATB multigene in soybean. Plants (Basel). 2021;10(11):2542. 34834905 10.3390/plants10112542 PMC8622832

[35] Liu JH, Zhang J, Jia CH, Zhang JB, Wang JS, Yang ZX, Xu BY, Jin ZQ. The interaction of banana MADS-box protein MuMADS1 and ubiquitin-activating enzyme E-MuUBA in post-harvest banana fruit. Plant Cell Rep. 2013;32(1):129–37. 23007689 10.1007/s00299-012-1347-4

[36] Gupta R. Analysis of cellulose degrading enzymes in genetically modified sugarcane juice using capillary electrophoretic techniques[D]. University of Manchester; 2017.

[37] Dima Kozakov D, Hall DR, Xia B, Porter KA, Padhorny D, Yueh C, Beglov D, Vajda S. The cluspro web server for protein–protein Docking. Nat Protoc. 2017;12:255–78. 28079879 10.1038/nprot.2016.169 PMC5540229

[38] Xing HL, Dong L, Wang ZP, Zhang HY, Han CY, Liu B, Wang XC, Chen QJ. A CRISPR/Cas9 toolkit for multiplex genome editing in plants. BMC Plant Biol. 2014;14:327. 25432517 10.1186/s12870-014-0327-y PMC4262988

[39] Zd’Arska M, Zatloukalova P, Benitez M, et al. Proteome analysis in Arabidopsis reveals shoot- and root-specific targets of cytokinin action and differential regulation of hormonal homeostasis[J]. Plant Physiol. 2013;161(2):918–30. 23209126 10.1104/pp.112.202853 PMC3561029

[40] Emily N, Kennedy SD, Hebdon SK, Menon CA, Foster DM, Copeland. Qingping xu,fabiola Janiak-Spens, ann H. West. Role of the highly conserved G68 residue in the yeast phosphorelay protein Ypd1:implications for interactions between histidine phosphotransfer (HPt) and response regulator proteins BMC biochemistry. 2019;20:12–4. 10.1186/s12858-019-0104-5 PMC6341664 30665347

[41] Ferreira FJ, Kieber JJ. Cytokinin signaling[J]. Curr Opin Plant Biol. 2005;8(5):518–25. 16054432 10.1016/j.pbi.2005.07.013

[42] Benjamin J, Frank L, Alexander S, et al. Identification of the transporter responsible for sucrose accumulation in sugar beet taproots. Nat Plants. 2015;1(1):14001. 27246048 10.1038/nplants.2014.1

[43] Han Y, Han S, Ban Q, et al. Overexpression of persimmon DkXTH1 enhanced tolerance to abiotic stress and delayed fruit softening in Transgenic plants[J]. Plant Cell Rep. 2017;36(4):583–96. 28155115 10.1007/s00299-017-2105-4

[44] Xie L, Yang C, Wang X. Brassinosteroids can regulate cellulose biosynthesis by controlling the expression of CESA genes in Arabidopsis[J]. J Exp Bot. 2011;62(13):4495–506. 21617247 10.1093/jxb/err164 PMC3170551

[45] Kaltdorf M, Dandekar T, Naseem M. Reconstruction of an immune dynamic model to simulate the contrasting role of auxin and cytokinin in plant immunity[J]. Methods Mol Biol. 2017;1569:83–92. 28265989 10.1007/978-1-4939-6831-2_6

[46] Boatwright JL, Pajerowska-Mukhtar K. Salicylic acid: an old hormone up to new tricks[J]. Mol Plant Pathol. 2013;14(6):623–34. 23621321 10.1111/mpp.12035 PMC6638680

[47] Bannenberg G, Martinez M, Hamberg M, et al. Diversity of the enzymatic activity in the Lipoxygenase gene family of *Arabidopsis thaliana*. Lipids. 2009;44(2):85–95. 18949503 10.1007/s11745-008-3245-7

[48] Dewitte W, Scofield S, Alcasabas AA, et al. Arabidopsis CYCD3 D-type cyclins link cell proliferation and endocycles and are rate-limiting for cytokinin responses[J]. PNAS. 2007;104(36):14537–42. 17726100 10.1073/pnas.0704166104 PMC1964848

[49] Jurgens G. Cytokinesis in higher plants. Annu Rev Phytopathol. 2005;56:281–99. 10.1146/annurev.arplant.55.031903.141636 15862097

[50] Sozzani R, Maggio C, Giordo R, et al. The E2FD/DEL2 factor is a component of a regulatory network controlling cell proliferation and development in Arabidopsis[J]. Plant Mol Biol. 2010;72(4–5):381–95. 19937368 10.1007/s11103-009-9577-8

[51] Fu X, Ng C, Feng D, et al. Cdc48p is required for the cell cycle commitment point at start via degradation of the G1-CDK inhibitor Far1p[J]. J Cell Biol. 2003;163(1):21–6. 14557244 10.1083/jcb.200307025 PMC2173437

