# Supplementary material for: BvHP4b gene in red beet promotes tuber enlargement and enhances resistance to Pst DC3000
Source: BMC Genomics. 2025 Aug 7;26:731. doi: 10.1186/s12864-025-11864-8 (PMC12330197; doi:10.1186/s12864-025-11864-8)
Supplement: Supplementary file 1 — Supplementary Material 1. [file 12864_2025_11864_MOESM1_ESM.pdf]

Figure S1: Analysis of protein expression in transgenic strains

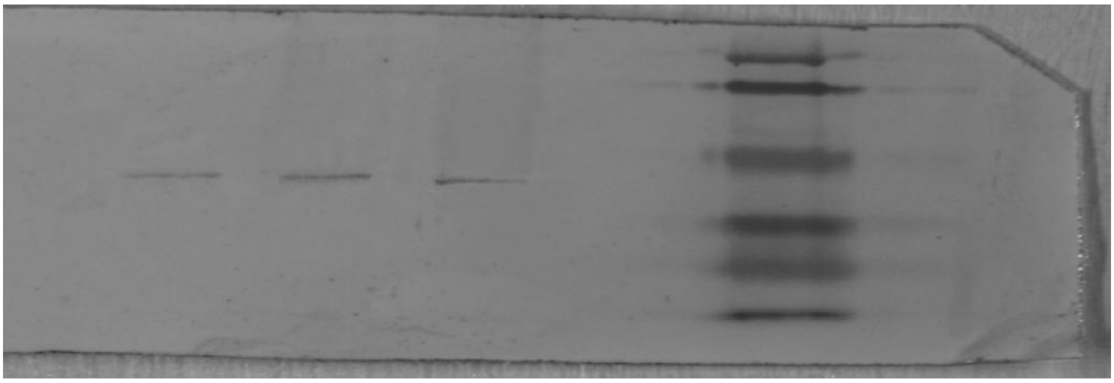

Protein expression of transgenic Arabidopsis ectopic expression BvHP4b, The molecular weight of BvHP4b-GFP fusion protein is 45.2kDa

Figure S2: Construction of yeast two hybrid vector

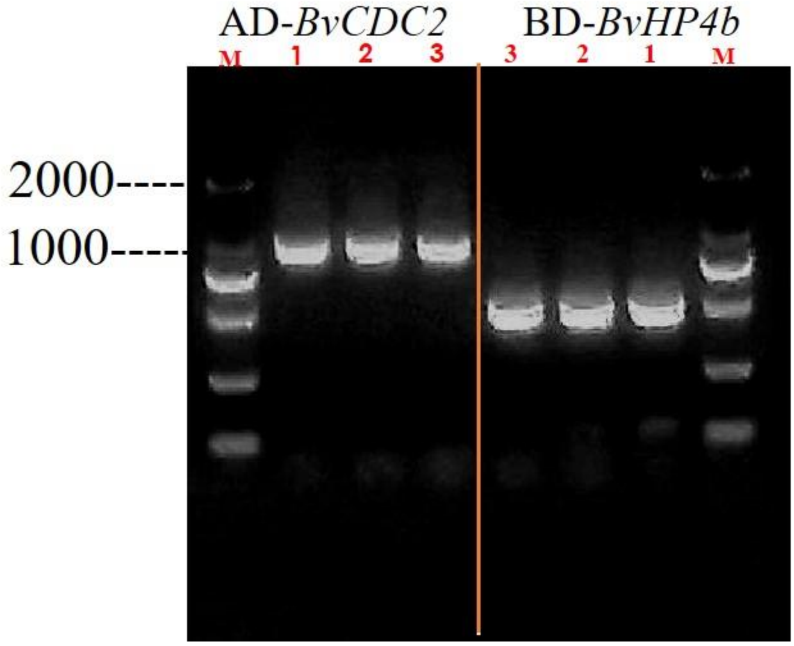

Legend: AD BvCDC2 and BD BvHP4b vectors were successfully constructed by gel electrophoresis, DM=2000
